# Application of a Mixed-Ligand Metal–Organic Framework in Photocatalytic CO_2_ Reduction, Antibacterial Activity and Dye Adsorption

**DOI:** 10.3390/molecules28135204

**Published:** 2023-07-04

**Authors:** Hongwei Jing, Lun Zhao, Guanying Song, Jiayu Li, Ziyun Wang, Yue Han, Zhexin Wang

**Affiliations:** College of Chemistry, Changchun Normal University, Changchun 130032, Chinaqx202000034@stu.ccsfu.edu.cn (Z.W.)

**Keywords:** mixed ligand, photocatalytic CO_2_ reduction, antibacterial, dye adsorption

## Abstract

In this paper, a known mixed-ligand MOF {[Co_2_(TZMB)_2_(1,4-bib)_0.5_(H_2_O)_2_]·(H_2_O)_2_}_n_ (compound **1**) was reproduced, and its potential application potential was explored. It was found that compound **1** had high photocatalytic activity for CO_2_ reduction. After 12 h of illumination, the formation rate of CO, which is the product of CO_2_ reduction by compound **1**, reached 3012.5 μmol/g/h. At the same time, compound **1** has a good antibacterial effect on *Staphylococcus aureus* (*S. aureus*), *Escherichia coli* (*E. coli*) and *Candida albicans* (*C. albicans*), which has potential research value in the medical field. In addition, compound **1** can effectively remove Congo Red from aqueous solutions and achieve the separation of Congo red from mixed dye solutions.

## 1. Introduction

A porous solid material is formed by the self-assembly of an inorganic metal ion (or cluster) with some organic ligand, which we define as a metal–organic framework, or MOF. Generally, in metal–organic skeletal materials, there is an interaction between the metal and the organic ligand, which is also referred to as a coordination polymer, CP. MOFs can be synthesized by selecting organic ligands containing different atoms such as O, N, P and S, changing the central metal atom and coordination mode, and controlling other external environmental factors including solvent, temperature, pH, etc., to tune the network structure, shape and properties of the compound. In recent years, with the increasing number of synthesized MOFs, exploring its excellent performance in various fields has become the direction of recent researchers [1,2,3,4,5].

The CO_2_ levels in the atmosphere have increased dramatically due to phenomena such as industrial production and exhaust emissions, while at the same time, the human exploitation of nature has reduced the absorption of CO_2_ by trees, thus exacerbating the greenhouse effect. The natural way to consume CO_2_ is through photosynthesis in green plants, and photocatalytic CO_2_ reduction is the closest redox reaction to natural photosynthesis. This technology converts CO_2_ into energy products such as CH_4_, CH_3_OH and CH_3_COOH through photocatalytic reactions. It has the advantages of being green and efficient, having a low energy consumption and having no secondary pollution, thus becoming an ideal solution to environmental pollution. As might be expected from photosynthesis, photocatalytic reduction systems require substances that absorb light (photosensitizers), substances that provide electrons (electronic sacrificial agents) and substances that enable the catalytic reaction to proceed efficiently (catalysts). The catalysts that have been reported for use in photocatalytic CO_2_ reduction systems are semiconductor materials, organic photosensitive dyes and precious metal compounds. However, their application is limited by their low light absorption efficiency, high price and lack of availability. Metal–organic frameworks (MOFs) are porous and have a large specific surface area, which allows for the introduction of visible-light-responsive ligands and tunable metal sites in their synthesis, and enables MOFs to be used as photocatalyst materials for the photoreduction of carbon monoxide [6,7,8]. In photocatalytic reaction, MOFs play a similar role to traditional semiconductors, but multiple sites such as organic ligands and metal clusters in MOFs can cooperate to enhance the reaction [9,10]. Han and workers synthesized a series of composites. Among them, TPVT-MOFs@g-C_3_N_4_-10 was used for the photocatalytic reduction of CO_2_ and the oxidation of H_2_O, and the yield could reach 56.4 μmol/g/h, which was 3.2 times higher than that of pure g-C_3_N_4_ (17.5 μmol/g/h) [11]. Additionally, Zhang and researchers synthesized NiFe_2_O_4_@N/C/SnO_2_ for use as nanometer photocatalysts. The results of the photocatalytic experiments show that the catalyst samples annealed at 450 °C gave the highest CO yield of 2057.41 μmol/g/h from CO_2_ [12].

As the most abundant organism, bacteria have been threatening human life. Bacteria can be classified as Gram-positive (*S. aureus*) or Gram-negative (*E. coli*) based on Gram staining results. Fungi are eukaryotic organisms and can cause diseases such as candidiasis and aspergillosis [13]. The discovery and use of antibiotics is an important milestone in the history of human medicine, but the drug resistance of pathogenic bacteria caused by its abuse has seriously threatened human life, health and safety. The latest research found that MOF, as a porous material, has a great development space in the biomedical field based on its special structural skeleton. It can improve biocompatibility and reduce biotoxicity by regulating metal nodes or organic bridging ligands, and can play an antibacterial role against sensitive and drug-resistant microorganisms [14,15,16,17,18]. Lu and investigators synthesized two Ag-MOFs and tested them against the Gram-negative bacterium *Escherichia coli* (*E. coli*) and the Gram-positive bacterium *Staphylococcus aureus* (*S. aureus*). The experiments showed that MOFs can rupture bacterial membranes and cause cell death. Hematological studies showed that MOFs exhibited good biocompatibility in mice [19]. Shams and team members synthesized Cu/H_3_BTC MOF and tested it as an antibacterial agent against *Staphylococcus aureus* (*S. aureus*) and *Escherichia coli* (*E. coli*). The results show that it could disrupt the cell membrane, which led to the expulsion of cellular components. The antibacterial effect of Cu/H_3_BTC MOF on *S. aureus* was better than that of *E. coli*. The minimum inhibitory concentration test and growth curve test of Cu/H_3_BTC MOF also proved its excellent antibacterial properties. And the results of agarose gel electrophoresis (AGE) show that Cu/H_3_BTC MOF could enter the bacterial cells and inhibit DNA synthesis by disrupting cell membranes [20]. Nakhaei and team chose Zn(NO_3_)_2_-6H_2_O and benzene-1-4-dicarboxylic acid (H_2_BDC) to synthesize three structurally different MOFs (MOF-5, Zn-MOF, TMU-3) under different conditions and analyzed their antibacterial properties using the agar diffusion method. For *S. aureus*, the diameter of the inhibition circle was Zn-MOF (15.5 mm) > MOF-5 (10 mm) = TMU-3 (10 mm), with a stronger inhibition of the active sample (Ac Zn-MOF (16 mm) = Ac MOF-5 (16 mm) > Ac TMU-3 (14 mm)). For *E. coli*, the inhibition circle diameter was Zn-MOF (14 mm) > TMU-3 (10 mm) > MOF-5 (0 mm), and the inhibition was similarly enhanced for the active sample (Ac Zn-MOF (14 mm) > Ac TMU-3 (12 mm) > Ac MOF-5 (10 mm)) [21].

Common adsorption materials mainly include molecular sieve, activated carbon and other porous materials [22,23,24]. MOFs are also porous materials, as the above materials are the same, so some of them have good adsorption performance [25,26,27,28]. Because of their high specific surface area and good thermal stability, the MOFs can be used for the adsorption and separation of organic dye molecules so as to solve the separation of MOF substances in liquid and the purification of different components, which is also a major subject of material science research at present. Liu and researchers synthesized one CoFe-MOF with an adsorption capacity of 1935.68 mg/g for Congo red, while the adsorption capacities of the MOF materials containing Fe and Co monometals were 775.19 and 628.93 mg/g, respectively, indicating that the CoFe-MOF has more defects, and thus, a higher adsorption efficiency [29]. Farhad et al. used UiO-66 to examine its ability to separate four contaminating dyes via the adsorption of methyl red (MR), methyl orange (MO), malachite green (MG) and methylene blue (MB), and studied the structural stability of the adsorbent in water, chloroform and dimethylformamide (DMF) over a period of one year, and the results show that UiO-66 is a chemically stable MOF. It was successfully synthesized and used for the removal of anionic and cationic dyes from aqueous solutions [30]. The study by Kumar et al. covers the application of MOFs in wastewater treatment/purification, aiming to address the treatment of the most persistent chemicals in water. The applicability of MOFs was evaluated for dynamic and low-cost wastewater treatment/purification options in combination with their advanced functions (adsorption, photodegradation/catalysis, separation and sensing). In order to further improve the practicality of MOF technology in WWT, significant efforts were invested in verifying its capacity and reliability for the treatment of various organic pollutants in water [31]. Zhan and researchers carried out adsorption experiments using an isotopically structured zirconium-based metal–organic backbone (Zr-MOF), which can be used for adsorption studies due to its good stability at high temperatures and pressures, as well as its chemical stability to acids and bases. The adsorption performance of Zr-MOF depends on the pore size of the MOF and the surrounding environment. In this study, the Zr-MOF material remained stable as the size changed and the adsorption proceeded, reaching an optimum adsorption state at 235 mg/g. During the adsorption process, physical adsorption occurs on the MOF surface, while chemisorption occurs in the form of dye molecules binding to the active site. It was eventually concluded that the larger the pore size, the greater the adsorption capacity, which is a contribution that provides new ideas for wastewater treatment problems [32].

In this paper, a known mixed-ligand MOF ({[Co_2_(TZMB)_2_(1,4-bib)_0.5_(H_2_O)_2_]·(H_2_O)_2_}_n_) [1] (compound **1**) was reproduced, and its potential application potential was explored. It was found that the compound has a good performance of the CO_2_ photocatalytic reduction of CO. Under the optimal reaction conditions after 12 h of illumination, the formation rate of CO reaches 3012.5 μmol/g/h. Through the antibacterial test, it can be found that compound **1** completely inhibits the growth of *S. aureus*, *E. coli* and *C. albicans* within 24 h. In addition, compound **1** can effectively remove Congo Red from aqueous solutions and achieve the separation of Congo red from mixed dye solutions.

## 2. Results and Discussion

### 2.1. Photocatalytic CO_2_ Reduction

**Figure 1:**
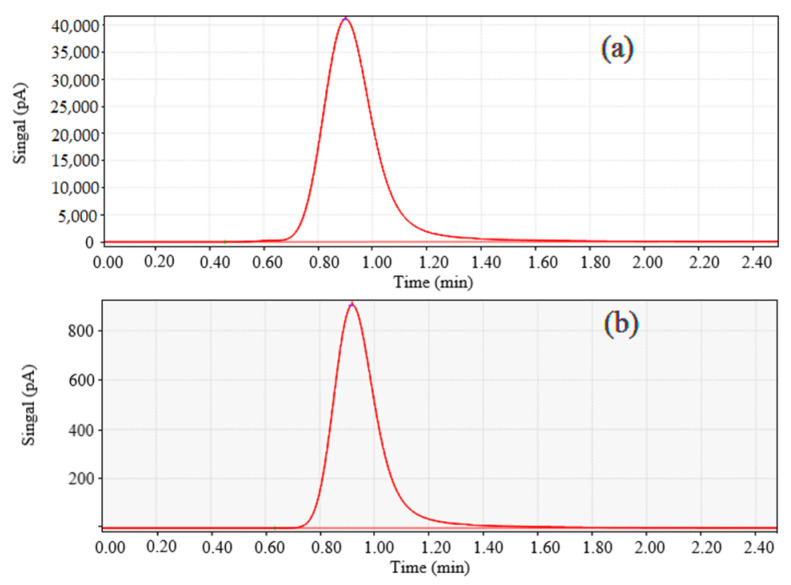
Retention time of (**a**) standard CO, (**b**) reduction product in gas chromatography.

The nature of photocatalytic CO_2_ reduction is a redox reaction in which CO_2_ is reduced to HCOOH, CH_3_OH or gaseous products such as CO, CH_4_, H_2_ and C_2_H_4_ in the presence of a reducing agent. The reaction products were qualitatively detected via gas chromatography as CO (Figure 1). The choice of solvent is critical to the reaction system, and the solubility of the compound in the solvent will directly affect the CO_2_ reduction efficiency. In this paper, three reagents, DMF, DMA and CH_3_CN, were chosen to be mixed with water as reaction solvents. All three solvents have a good solubility and stability for a variety of substances. The pressure of the photocatalytic reduction reaction system was 1 MPa with compound **1** as catalyst, bipyridylruthenium (Ru) as a photosensitizer and TEOA as an electron sacrificial agent.

**Figure 2:**
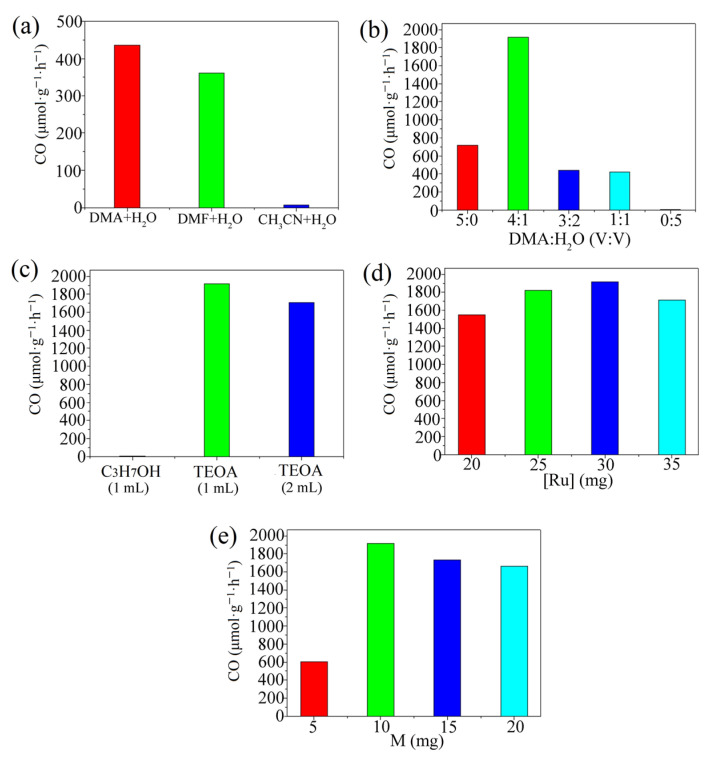
(**a**) CO production rate for different systems; (**b**) CO generation rates for different solvent ratios; (**c**) CO generation rates for different types and ratios of electron sacrificial agents; (**d**) CO generation rate at different photosensitizer dosages; (**e**) CO generation rates for different amounts of complexes.

Further, experiments were carried out regarding the ideal type of reaction solvent and its ratio, the ideal type and amount of electron sacrificial agent, the ideal amount of photosensitizer and the ideal amount of compound (Figure 2). The experimental results show that 10 mg of compound **1**, 30 mg of photosensitizer (Ru) and 6 mL of solvent (DMA:H_2_O:TEOA = 4:1:1) were the optimum reaction conditions for the reaction system.

According to the optimum reaction conditions, the photocatalytic reaction was carried out. Firstly, carbon dioxide was continuously injected into the top-illuminated photocatalytic reactor to eliminate the interference of impurities. After about 15 min, we closed the valve and continued to introduce CO_2_ so that the pressure in the reaction kettle could reach 1 MPa. The PLS-SXE300D xenon lamp with a 420 nm filter was irradiated continuously for 12 h, and the samples were analyzed via gas chromatography every 3 h, and the cycle was repeated more than three times to prove its accuracy.

**Figure 3:**
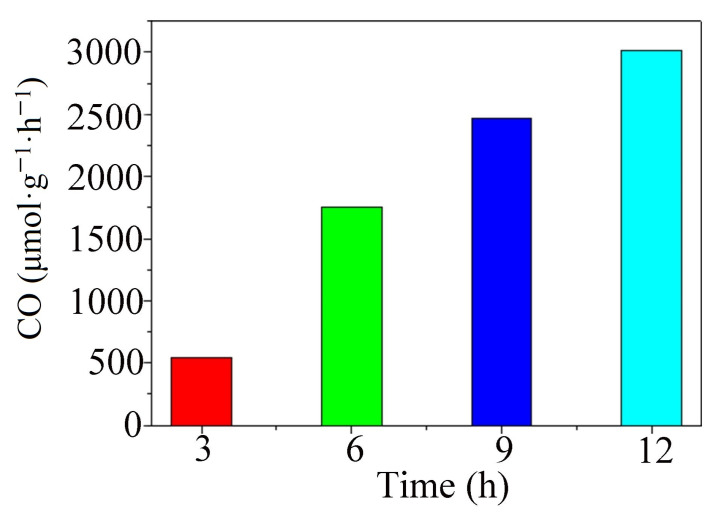
The rate of CO production at different time intervals.

It is easy to see from Figure 3 that the CO production rate increased linearly with time. The rate of product formation per unit mass of sample was calculated using the formula PV = nRT, and the product CO produced by the photocatalytic reduction of carbon dioxide in compound **1** was as high as 3012.5 μmol/g/h (P = 1 MPa, T = 313.15 K, R = 8.31 J/(mol·K), V was calculated from the ratio of the real-time peak area of the product to the peak area of 1 mL of standard gas).

After that, the control test was carried out for compound **1**. The results show that the formation rate of CO is negligible without the presence of complexes, (Ru) and TEOA, respectively (Table 1). In dark conditions and without CO_2_, CO will not be generated. This shows that each component in the system is indispensable for the photocatalytic reduction of CO_2_, and the existence of compound **1** greatly increases the rate of CO production, indicating that compound **1** plays a crucial role in the reaction system. After the experiment, the recovered compound **1** was analyzed via powder XRD, and the results show that compound **1** had good stability.

### 2.2. Antibacterial Properties

In this paper, *S. aureus* (ATCC 6538), *E. coli* (CMCC 44102) and *C. albicans* (ATCC 10231) were selected as experimental objects. The experimental instruments, prepared LB medium and PDB medium were sterilized with high-pressure steam and transferred to an ultra-clean table. After inoculation, they were cultured in a shaker at 37 °C. After leaving it overnight, the bacterial liquid was diluted 100 times with the culture medium for later use. We prepared a certain amount of solid culture medium. A proper amount of *S. aureus* and *E. coli* diluted to 10^7^ CFU/mL were coated in LB medium, and *C. albicans* were coated in PDB medium evenly. After the bacterial liquid was absorbed, the circular paper was soaked with different concentrations (1 mg/mL, 5 mg/mL) of the compound and then put into the culture medium. Blank and heterocyclic multidentate carboxylic acid ligands were used as the controls. The treated plate was cultured in a constant temperature incubator at 37 °C for 24 h. We then observed and recorded the diameter of the suppression ring.

**Figure 4:**
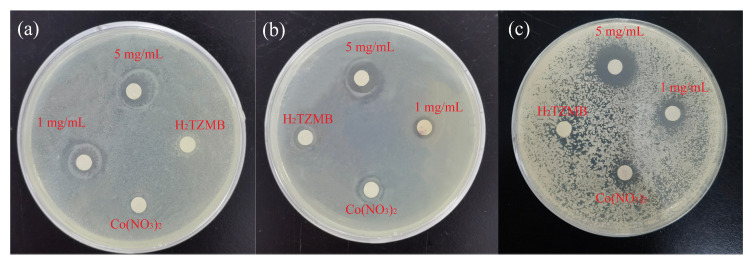
(**a**) Inhibition zone of MOF against (**a**) *S. aureus*, (**b**) *E. coli* and (**c**) *C. albicans*.

The control experiment showed that metal salts and mixed ligands had no antibacterial effect on the bacterial solution. When the concentration of the solution of compound **1** was 5 mg/mL, the diameter of the bacteriostatic circle for *S. aureus* reached 14 mm, and the minimum inhibitory concentration MIC was 500 μg/mL (Figure 4a). For *E. coli*, the diameter of the bacteriostatic circle was 11 mm, and the MIC was 400 μg/mL (Figure 4b). For *C. albicans*, the diameter of the bacteriostatic circle was 11 mm, and the MIC was 200 μg/mL (Figure 4c).

**Figure 5:**
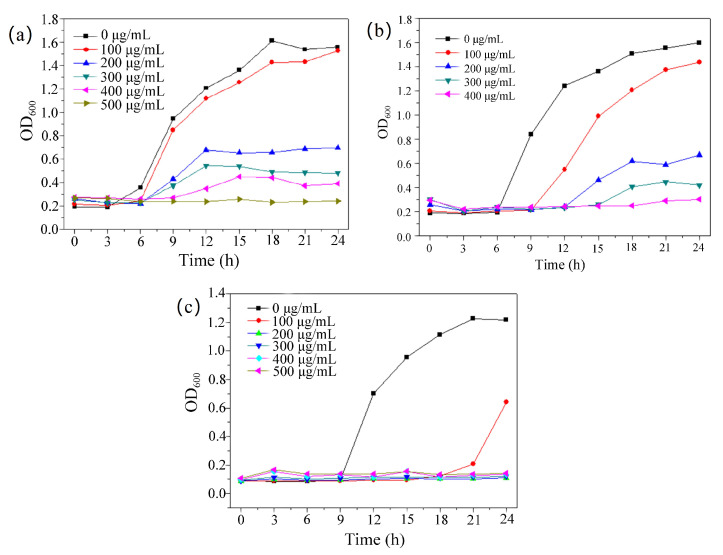
(**a**) Growth curve of MOF against *S. aureus*; (**b**) growth curve of MOF against *E. coli*; (**c**) growth curve of MOF against *C. albicans*.

We prepared different concentrated solutions of compound **1** in the LB medium and added appropriate amounts of diluted *E. coli* and *S. aureus*, respectively; we prepared different concentrated solutions of compound **1** in the PDB medium and added appropriate amounts of diluted *C. albicans*. The bacterial solution was placed in a shaker at 37 °C, and the absorbance at 600 nm (OD600) was measured every 3 h using an enzyme marker to obtain the growth curve of the strains. It can be seen from the growth curve that the bacterial solution of the blank control group increased exponentially. For *S. aureus* (Figure 5a), with the increase in the concentration of compound **1**, the bacteriostatic effect was gradually enhanced. When the concentration of the complex was 500 μg/mL, the growth of the bacterial solution was completely inhibited. For *E. coli* (Figure 5b), the growth of the bacterial solution was also completely inhibited when the concentration of **1** reached 400 μg/mL. For *C. albicans* (Figure 5c), when the concentration of **1** was 200μg/mL, the growth of the bacterial solution was completely inhibited.

### 2.3. Dye Adsorption

Currently, with the rapid development of the economy, more and more environ-mental pollution problems have come along, among which dye pollution in water is a big aspect. Since compound **1** was very stable in the aqueous solution and had large pores in its structure, three organic dyes were selected to test its dye adsorption performance, which were Congo Red (CR), Methylene Blue (MB) and Rhodamine B (RhB).

**Figure 6:**
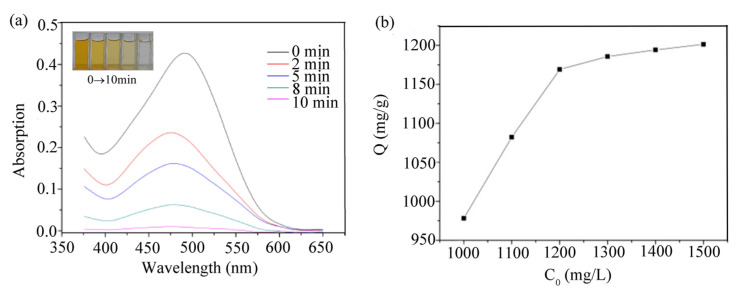
(**a**) UV–Vis spectra of CR with compound **1** at different time intervals (insert: photographs of dye solutions for given period of time); (**b**) adsorption isotherms for CR adsorption over 1 h (compound **1**). C_0_: the initial concentration of adsorbate, Q: the amount of CR adsorbed.

First, 10 mg of compound **1** was added to 50 mL (5 × 10^−5^ mol·L^−1^) of the dye aqueous solution. Subsequently, the UV spectra of the mixture at different time intervals were recorded after the mixture reached the adsorption dissociation equilibrium. The UV spectral analysis in Figure 6a and the photos before and after adsorption show that compound **1** has a fast adsorption capacity for CR, while it has almost no adsorption capacity for the other two dyes. In ten minutes, the removal of CR by compound **1** can reach 99% (Equation (S1), ESI†).

Through the adsorption capacity experiment (Figure 6b), it can be found that the adsorption capacity of compound **1** to CR can reach 1194 mg·g^−1^ (Equation (S2)). The unique adsorption capacity of compound **1** is mainly due to the electrostatic interaction between its pores and CR molecules. A release test was also carried out to confirm the regeneration capacity of the adsorbent. Compound **1** was placed in a methanol solution and released after the adsorption of CR (5 × 10^−5^ mol·L^−1^). For compound **1**, the CR was released with an efficiency of 99% (Figure S8, ESI†). The XRD data show that the structure of compound **1** did not change before and after the experiment (Figure S9, ESI†). This indicates that compound **1** has good regeneration performance during the adsorption–desorption process.

In addition, the UV–Vis spectroscopic analysis and before and after photos of the adsorption reveal that compound **1** showed almost no adsorption of the cationic dyes of MB and RhB (Figure S10, ESI†).

**Figure 7:**
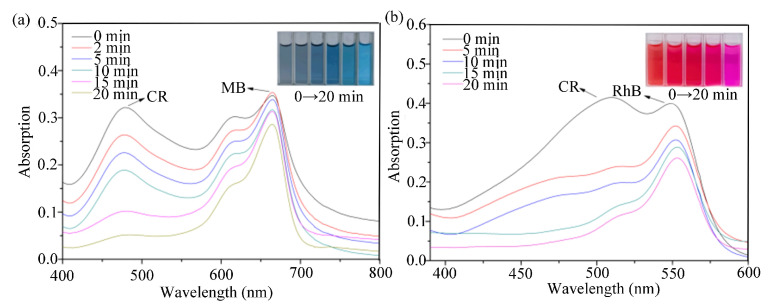
UV–Vis spectra of mixed dye solution at different time intervals: (**a**) **1**@CR/MB, (**b**) **1**@CR/RhB (insert: photographs of dye solutions for given period of time).

To further investigate the selective adsorption ability of compound **1** on CR, CR/MB and CR/RhB were mixed to obtain brown and pink solutions, respectively, and 10 mg of compound **1** was added. The solutions showed blue and pink colors after 20 min, respectively, indicating an effective selective adsorption of the mixed dyes by compound **1**. The CR removal reached 84% (CR/MB) and 85% (CR/RhB) (Figure 7a,b). 

**Figure 8:**
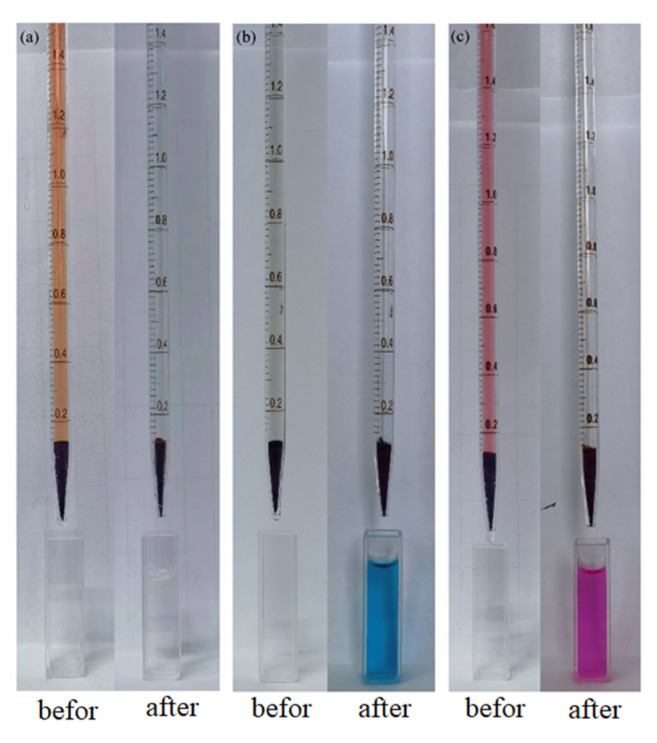
Column chromatography experiments using compound **1** (100 mg) as a filter: (**a**) **1**@CR, (**b**) **1**@CR/MB, (**c**) **1**@CR/RhB.

The above experiments demonstrated the potential selective separation ability of compound **1** for the CR dye, and it was therefore used as a stationary phase for the column to more clearly express its ability to effectively remove CR from aqueous solutions. In the experiment, a pipette was used to simulate the chromatographic column, and 100 mg of compound **1** was filled in it to filter the mixed solutions of CR, CR/MB and CR/RhB. As shown in Figure 8, after filtration, the CR dyes were all adsorbed by compound **1**, with the single dyes showing no color, and the mixed dyes showing another color. Thus, compound **1** shows a potential application in the efficient separation of CR from mixed dye solutions.

## 3. Materials and Methods

### 3.1. Materials and Instrumentation

Powder X-ray diffraction (PXRD) data were recorded on the D2 PHASER A26-X1 XRD diffractometer. The IR spectra (4000–400 cm^−1^) were obtained from KBr pellets with an FTIR Nexus spectrophotometer. Elemental analyses were performed on a PerkinElmer 240 C analyzer. Spectra Max Plus 384 microplate reader was used to determine OD 600 of bacterial liquid. The ultraviolet absorption spectrum was collected using Cary 300 spectrophotometer. The composition and content of CO_2_ reduction products were recorded using the gas chromatograph (GC-7920).

### 3.2. Synthesis of MOF

In this paper, an MOF with the same structure was synthesized using a synthesis method similar to that used by the Hu team [1]. A mixture of Co(NO_3_)_2_·6H_2_O (32.1 mg, 0.1 mmol), 1,4-bib (21 mg, 0.1 mmol) and H_2_TZMB (32.3 mg, 0.1 mmol) was dissolved in 10 mL of H_2_O solvent (Figure S1, ESI†). After stirring, the mixture was sealed in a 25 mL Parr Teflon-lined stainless-steel autoclave under autogenous pressure and heated at 160 °C for three days. Then, after slow cooling to room temperature at 20 °C·h^−1^, large quantities of purple bulk crystals were obtained, and the crystals were filtered off, washed with absolute ethyl alcohol and dried under ambient conditions. The final yield collected was 54 wt% on H_2_TZMB ligand. Elemental analysis calcd. for C_23_H_15_O_4_N_5_Co (wt%): C, 57.04; H, 3.12; N, 14.46. Found: C, 57.69; H, 3.28; N, 14.17. FT-IR (KBr pellets, cm^−1^): 3114 (s), 2880 (w), 1607 (s), 1525 (s), 1395 (s), 1313 (w), 1133 (m),1071 (m), 963 (s), 837 (m), 774 (s), 650 (m), 546 (s), 472 (s). Figure S2 shows the interpenetrating structure of compound **1** (ESI†).

### 3.3. Powder X-ray Diffraction (PXRD)

**Figure 9:**
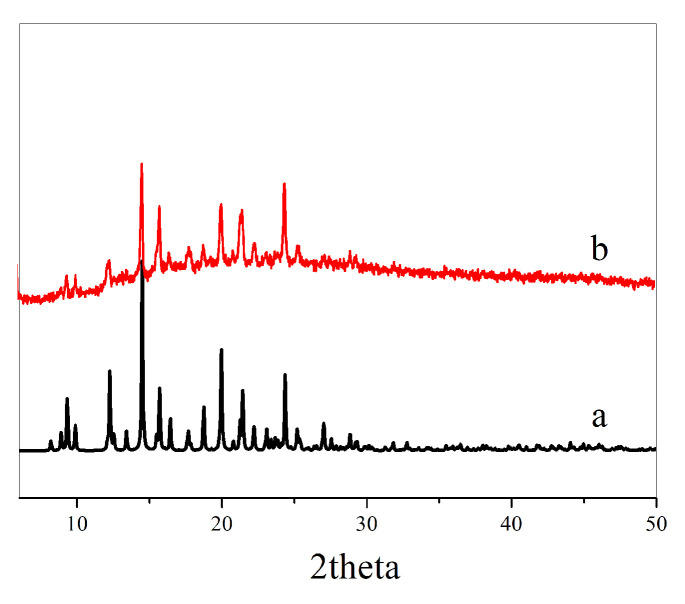
(**a**) XRD profile in the literature; (**b**) XRD profile of compound **1** synthesized using the method in the literature.

Compound **1** [1] was synthesized according to the synthetic route in the literature, and its XRD data were mildly similar to those in the literature, indicating that a high purity compound **1** was obtained (Figure 9).

### 3.4. Optimum Reaction Conditions of Photocatalysis

In the reaction system consisting of complex catalyst, photosensitizer, sacrificial agent and solvent, the reaction product is qualitatively detected as CO via gas chromatography. In order to test the optimal reaction conditions, the effects of different solvents and their proportions, the electron sacrificing agents and their proportions, the number of photosensitizers and the number of compounds on the reaction system were discussed in the experiment to determine the final experimental conditions. The above experiments determined the optimal reaction conditions of the reaction system to be the following: 10 mg of compound **1**, 40 mg of photosensitizer (Ru), 6 mL of solvent (DMA: H_2_O: TEOA = 4:1:1) and the CO_2_ pressure of 1 MPa. A controlled test was also carried out, and the results show that each component of the system was important for hanging photocatalytic CO_2_ reduction and that the addition of the complexes greatly increased the rate of CO production. We show the stability performance of compound **1** before and after the experiment by comparing the PXRD and TGA of compound **1** before and after CO_2_ reduction in Figures S3 and S4, ESI†.

### 3.5. Bacterial Incubation and Bacteriostatic Circle Experiment

The experimental apparatus and the configured LB medium and PDB medium were transferred to the ultraclean table after autoclaving, and then *S. aureus* (ATCC 6538), *E. coli* (CMCC 44102) and *C. albicans* (ATCC 10231) were incubated in a shaker at 37 °C. The overnight bacterial liquid was diluted 100 times with the culture medium for standby.

In the bacteriostasis circle experiment, appropriate amounts of *S. aureus* and *E. coli* diluted to 107 CFU/mL by the culture medium were evenly smeared in the LB culture medium, and *C. albicans* were smeared in the PDB culture medium. After the bacterial solution was absorbed, the round paper of the fully soaked compound **1** was put into the culture medium. The blank and mixed ligands were used as control. Then, we put them in a constant temperature incubator at 37 °C for 24 h and observed and recorded the diameter of the inhibition ring.

### 3.6. MIC Determination and Growth Curve Experiment

The bacterial solution after overnight culture was diluted to 10^5^ CFU/mL for standby. We diluted the compound **1** solution prepared with LB and PDB medium on a 96-well plate continuously (the volume was 100 μL), and added 100 μL of diluted bacterial solution. We put the treated pore plate in a constant temperature incubator at 37 °C, and obtained the MIC results after 24 h (Figures S5–S7, ESI†).

The LB medium was used to prepare different concentrations of the compound solution, and appropriate amounts of diluted *E. coli* and *S. aureus* were added, respectively. PDB medium was used to prepare different concentrations of compound solution, and an appropriate amount of diluted *C. albicans* was added. We put the bacterial solution into a shaker at 37 °C, and measured its absorbance at 600 nm wavelength with a microplate reader every 3 h to obtain the growth curve of the bacterial species.

## 4. Conclusions

In this paper, a known compound was synthesized, and its photocatalytic properties, antibacterial properties and dye adsorption properties were investigated. In the photocatalysis experiment, a novel photocatalysis carbon dioxide reduction system was successfully constructed. In 12 h, the average generation rate of CO can reach 3012.5 μmol/g/h. At the same time, the high concentration of compound **1** can completely inhibit the growth of *S. aureus*, *E. coli* and *C. albicans* within 24 h. In addition, at one hour, the adsorption capacity of compound **1** to CR can reach 1194 mg·g^−1^ and can achieve a rapid separation of CR in mixed dye solutions. The above experiments show that compound **1** has potential applications in photocatalytic CO_2_ reduction, solid antibacterial activity and dye adsorption. 

## Figures and Tables

**Table 1:** Control experiments under different conditions.

Entry	(MOF) mg	[{Ru(bpy)_3_}^2+^] mg	TEOA mL	CO μmol/g/h
1	10	30	1	1757.53
2	0	30	1	167.37
3	10	0	1	4.13
4	10	30	0	6.59
5 ^a^	10	30	1	0
6 ^b^	10	30	1	0

Reaction conditions: in CO_2_-saturated DMA:H_2_O (5 mL, V:V = 4:1) under 6 h of irradiation. ^a^ In the dark. ^b^ Under Ar(without CO_2_).

## Data Availability

Data are available upon request.

## Supplementary Materials

The following are available online at https://www.mdpi.com/article/10.3390/molecules28135204/s1, Figure S1. Molecular structure of ligands, Figure S2. (a) The coordination environment of the Co(II) ions in compound **1**. (b,c) View of 3D net structure. (d) View of 4-fold interpenetrated framework, Figure S3. XRD patterns of the MOF (a represents theoretical XRD, b represents experimental XRD, and c represents XRD after three cycles), Figure S4. TGA patterns of the MOF (a represents experimental TGA, and b represents TGA after three cycles), Figure S5. Changes of MOF solution with different concentration on *S. aureus* in 24 h, Figure S6. Changes of MOF solution with different concentration on *E. coli* in 24 h, Figure S7. Changes of MOF solution with different concentration on *C. albicans* in 24 h, Figure S8. Adsorption-desorption curve for CR (before: 5 × 10^−5^ CR; after: desorption for **1** by methanol), Figure S9. (a) XRD profile of compound **1** synthesized by the method in the literature; (b), after adsorption test for CR; (c), after releasing test for CR, Figure S10. UV-Vis spectra of (a) MB and (b) RhB with **1** at different intervals, respectively (Insert: photographs of dye solutions for given period of time).

## Sample Availability

Samples of the compounds are available from the authors.
