# Supplementary material for: Application of a Mixed-Ligand Metal–Organic Framework in Photocatalytic CO2 Reduction, Antibacterial Activity and Dye Adsorption
Source: Molecules. 2023 Jul 4;28(13):5204. doi: 10.3390/molecules28135204 (PMC10343588; doi:10.3390/molecules28135204)
Supplement: Supplementary file 1 [file molecules-28-05204-s001.zip › molecules-2456671-supplementary.pdf]

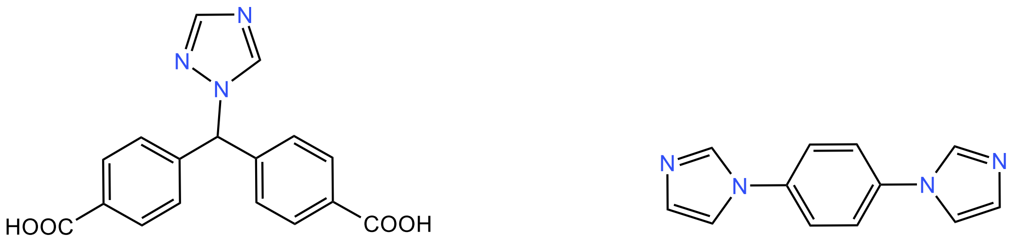

4,,4'-(1H-1,2,4-triazol-1-yl）methylenebis(benzonic acid) (H_2TZMB)

1,4-bis(1-imidazoly)benzene(bimb)

Figure S1. Molecular structure of ligands.

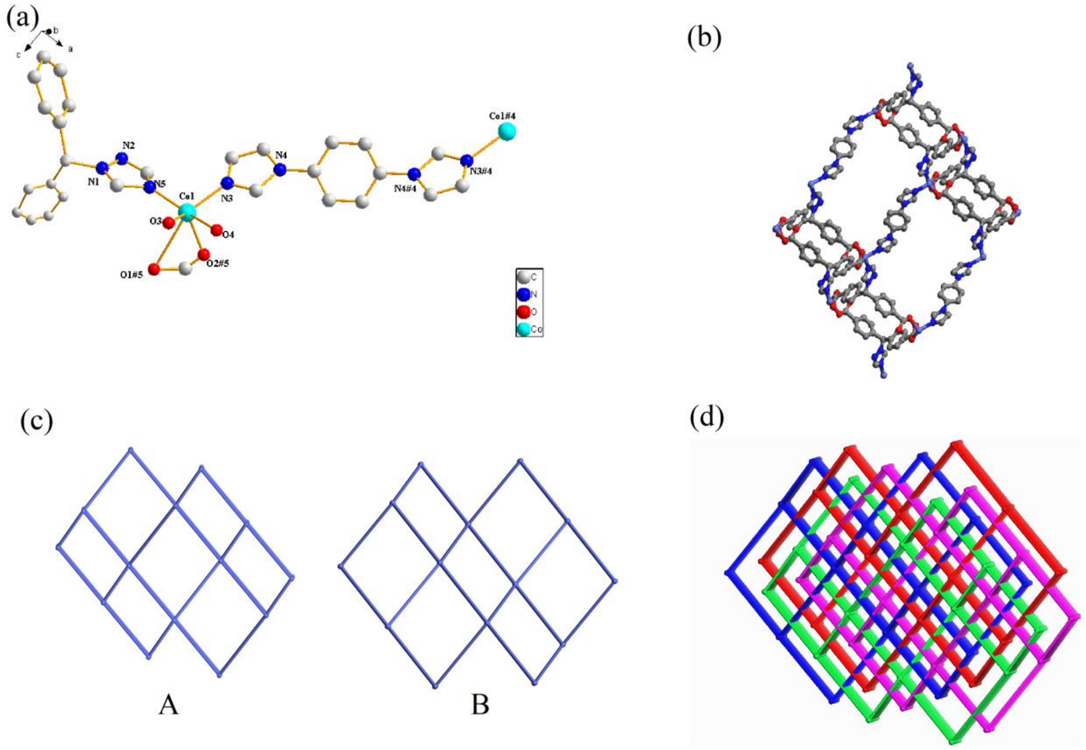

Figure S2. (a) The coordination environment of the Co(II) ions in compound **1**. (b,c) View of 3D net structure. (d) View of 4-fold interpenetrated framework.

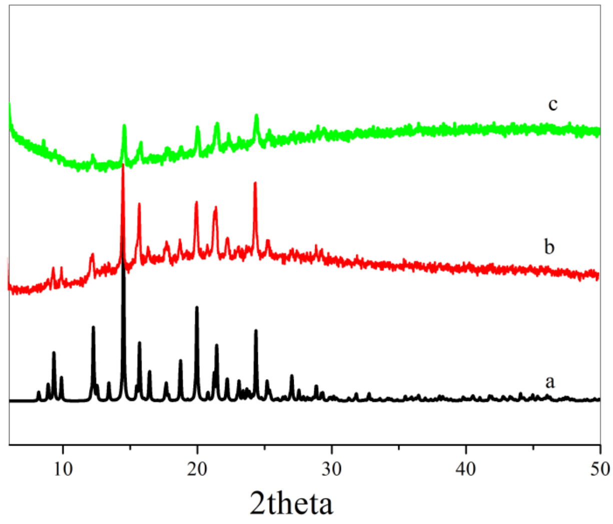

Figure S3. XRD patterns of the MOF (a represents theoretical XRD, b represents experimental XRD, and c represents XRD after three cycles).

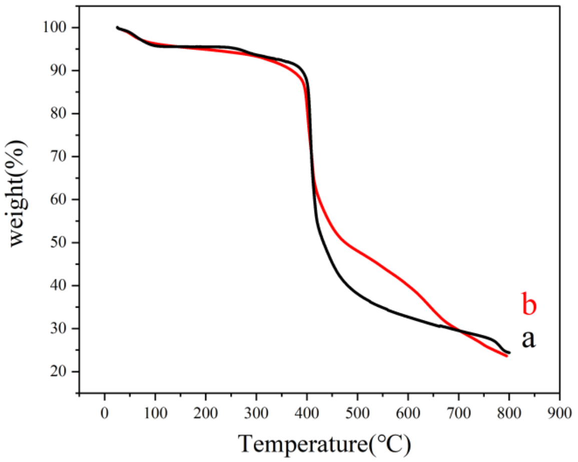

Figure S4. TGA patterns of the MOF (a represents experimental TGA, and b represents TGA after three cycles).

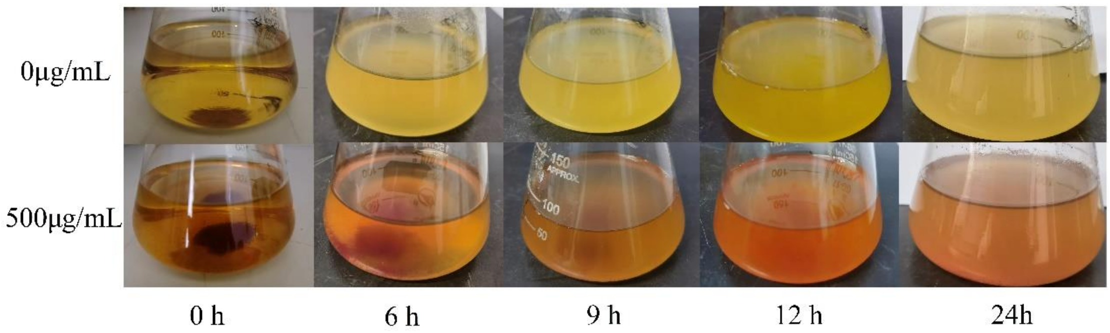

Figure S5. Changes of MOF solution with different concentration on S. aureus in 24 h.

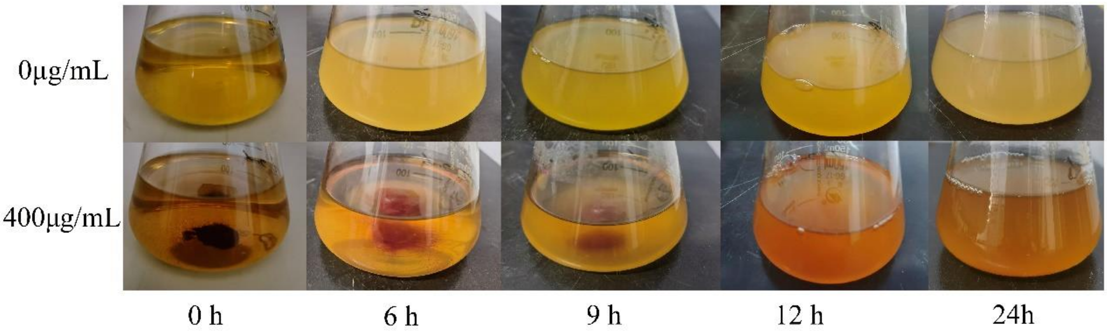

Figure S6. Changes of MOF solution with different concentration on E. coli in 24 h.

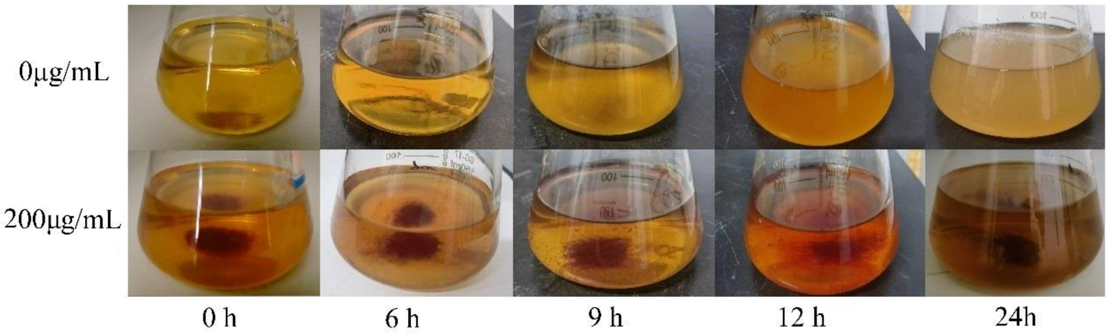

Figure S7. Changes of MOF solution with different concentration on C. albicans in 24 h.

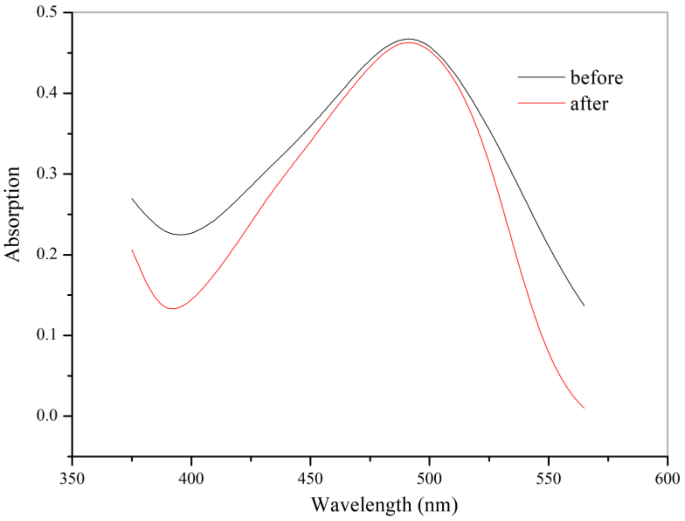

Figure S8. Adsorption-desorption curve for CR (before: 5×10^{-5} CR; after: desorption for **1** by methanol).

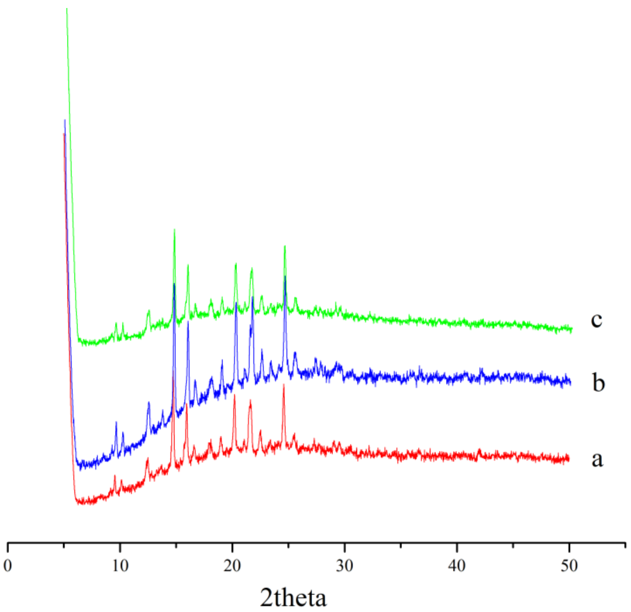

Figure S9. (a) XRD profile of compound **1** synthesized by the method in the literature; (b), after adsorption test for CR; (c), after releasing test for CR.

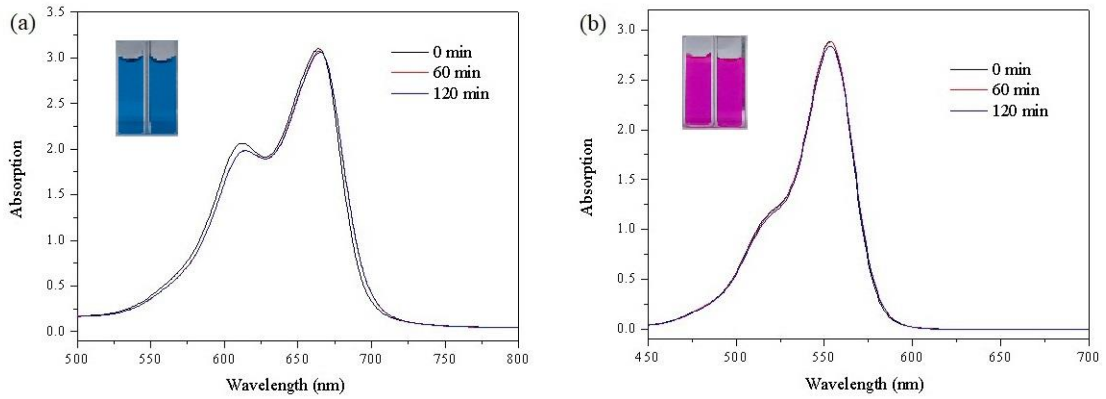

Figure S10. UV-Vis spectra of (a) MB and (b) RhB with **1** at different intervals, respectively (Insert: photographs of dye solutions for given period of time).

Equation:

The removal ratio (R) of compound **1** for dye was calculated using Equation (S1).

$$R = (C_0 - C_t) / C_0 \times 100\% \quad \text{(S1)}$$

The uptake of CR was calculated according to Equation (S2).

$$Q = (C_0 - C_e) \times V/m \quad \text{(S2)}$$

Where C_0 was the initial concentration (mg·L^{-1}), C_t was the solution concentration (mg·L^{-1}) at time t (min), C_e was the equilibrium solution concentration (mg·L^{-1}), Q (mg·g^{-1}) was the adsorption amount at different equilibrium solute concentration, V (L) was the volume of CR solution, m (mg) represented the mass of **1**.
